# Genotypes and drug resistance pattern of *Mycobacterium tuberculosis* complex among clinically diagnosed pulmonary tuberculosis patients

**DOI:** 10.3389/fpubh.2024.1420685

**Published:** 2024-12-02

**Authors:** Alem Alemayehu, Liya Wassie, Dawit Hailu Alemayehu, Bethlehem Adnew, Sebsib Neway, Dessalegn Abeje Tefera, Sosina Ayalew, Elena Hailu, Samuel Ayele, Berhanu Seyoum, Kidist Bobosha, Markos Abebe, Abraham Aseffa, Beyene Petros, Rawleigh Howe

**Affiliations:** ^1^Department of Microbial, Cellular and Molecular Biology, College of Natural and Computational Sciences, Addis Ababa University, Addis Ababa, Ethiopia; ^2^Armauer Hansen Research Institute (AHRI), Addis Ababa, Ethiopia; ^3^School of Medial Laboratory Science, College of Health and Medical Sciences, Haramaya University, Dire Dawa, Ethiopia

**Keywords:** BCPTB, CDPTB, Ethiopia, geneXpert, smear-microscopy, MTBC, spoligotyping, WGS

## Abstract

**Background:**

Clinically diagnosed pulmonary tuberculosis (TB) (CDPTB) patients account for a huge proportion of TB. However, little is known about the genetic diversity and drug resistance profile of *Mycobacterium tuberculosis* Complex (MTBC) strains in this group of patients.

**Method:**

Unmatched case–control study was conducted among 313 PTB patients to compare the genetic diversity of MTBC and their drug resistance profiles among CDPTB (*n* = 173) and bacteriologically confirmed pulmonary TB (BCPTB) (*n* = 140) patients. Lowenstein-Jensen (LJ) culture, geneXpert and acid fast staining were performed on sputum specimen collected from both CDPTB and BCPTB patients. Spoligotyping, whole genome sequencing (WGS) and phenotypic drug resistance testing (DST) were done for a subset of LJ grown MTBC isolates. Data was analyzed by STATA version 17 software and a *p*-value <0.05 were considered statistically significant.

**Results:**

The proportion of lineage 3 was larger among CDPTB patients (31%, 13/42) compared to BCPTB patients (15%, 11/74) (*p*-value <0.05). A higher proportion of MTBC isolates from CDPTB 16.6% (3/18) were phenotypically resistant to one or more anti-TB drugs than BCPTB 12% (4/33) (*p*-value >0.05). A single lineage 3 strain resistant to all the primary anti-TB drugs was detected in one CDPTB by both DST methods.

**Conclusion:**

The observed differences in the genotypes of MTBC isolates between CDPTB and BCPTB patients may be attributed to challenges in the identification of CDPTB that requires further investigation on sequenced genome of the MTBC strains for better understanding and recommendation based on the current finding. There was also primary drug resistant TB among culture positive CDPTB patients which would be otherwise missed by current national protocols.

## Introduction

Studying genetic diversity and drug resistance patterns of mycobacterial isolates is crucial to monitor the circulating *Mycobacterium tuberculosis Complex* (MTBC) strains in different communities for impactful control measures against disease transmission and deduce a better understanding of tuberculosis (TB) pathogenesis. Globally, clinically diagnosed pulmonary TB (CDPTB), also known as smear negative pulmonary TB (CDPTB) accounts for more than 40% of PTB and 35% of the PTB patient load in Ethiopia (1). Despite its high burden, there is scarcity of information on the genetic diversity and drug resistance characteristics of isolates from this form of TB.

CDPTB also known as smear-negative pulmonary TB (SNPTB) results in diagnostic delays and difficulty in monitoring treatment outcomes. Some of the challenges have been attributed to the limited availability of reagents for geneXpert or smear microscopy, lack of training on TB diagnostic tastings, lack of ideal antibacterial antibiotics for conventional bacterial pneumonia, as well as poor alignment with the WHO clinical algorithms for CDPTB (2). Less well appreciated are the unique microbiological features of the *Mycobacteria* and their genetic diversity, which could preclude detection of the bacilli under microscope.

Similarly, the overall emergence of drug-resistant (DR) or multi-drug resistant TB (MDR-TB) (resistant to at least rifampicin and isoniazid drugs), which also accounts for 17–33% in CDPTB patients (3, 4), imposes a huge burden on the TB control programs.

Recently, we have shown a direct relationship between disease severity, bacillary load and the outcome of laboratory diagnosis particularly, acid fast bacilli (AFB) positivity of PTB patients (5). Moreover, MTBC lineage types has been associated with disease severity, culture positivity and abundance of cell wall lipid metabolism proteins (6, 7) which all are interrelated and may contribute for pathogenesis and clinical outcome of TB diseases. Therefore, understanding differences in the lineage composition of MTBC complex as well as its drug resistance profiles in different clinical forms of TB is crucial to recognize TB pathogenesis and associated diagnostic challenges for better development of therapeutics and diagnostics to end TB. This study aimed to characterize the genetic diversity and drug resistance profiles of MTBC isolated from CDPTB patients relative to bacteriologically confirmed pulmonary TB (BCPTB) patients in Addis Ababa, Ethiopia.

## Methods

### Study setting

This work is a continuation of a recently published study that involved a cohort of CDPTB (who previously were known as smear-negative PTB patients) and BCPTB (who previously were known as smear-positive PTB patients) patients (5). Briefly, 173 clinically diagnosed and 140 bacteriologically confirmed PTB patients were recruited from health facilities in Addis Ababa with an unmatched case–control approach. According to the national TB diagnosis guideline (8), a total of 313 newly diagnosed, adult (age ≥ 18 years), PTB patients who attended TB clinics were enrolled in the study from selected health centers in Addis Ababa, Ethiopia. Patients who were initiated on anti-TB treatment for more than 5 days prior to enrolment, those who relapsed or were on retreatment, or those who were unable to provide sputum samples at the time of enrolment were excluded. Of the 313 PTB patients, 181 (58%) were diagnosed in public health facilities, 117 (37%) were from private health facilities, and the remaining 15 (5%) were identified through the community TB outreach system.

### Definition of cases and controls

**Figure 1:**
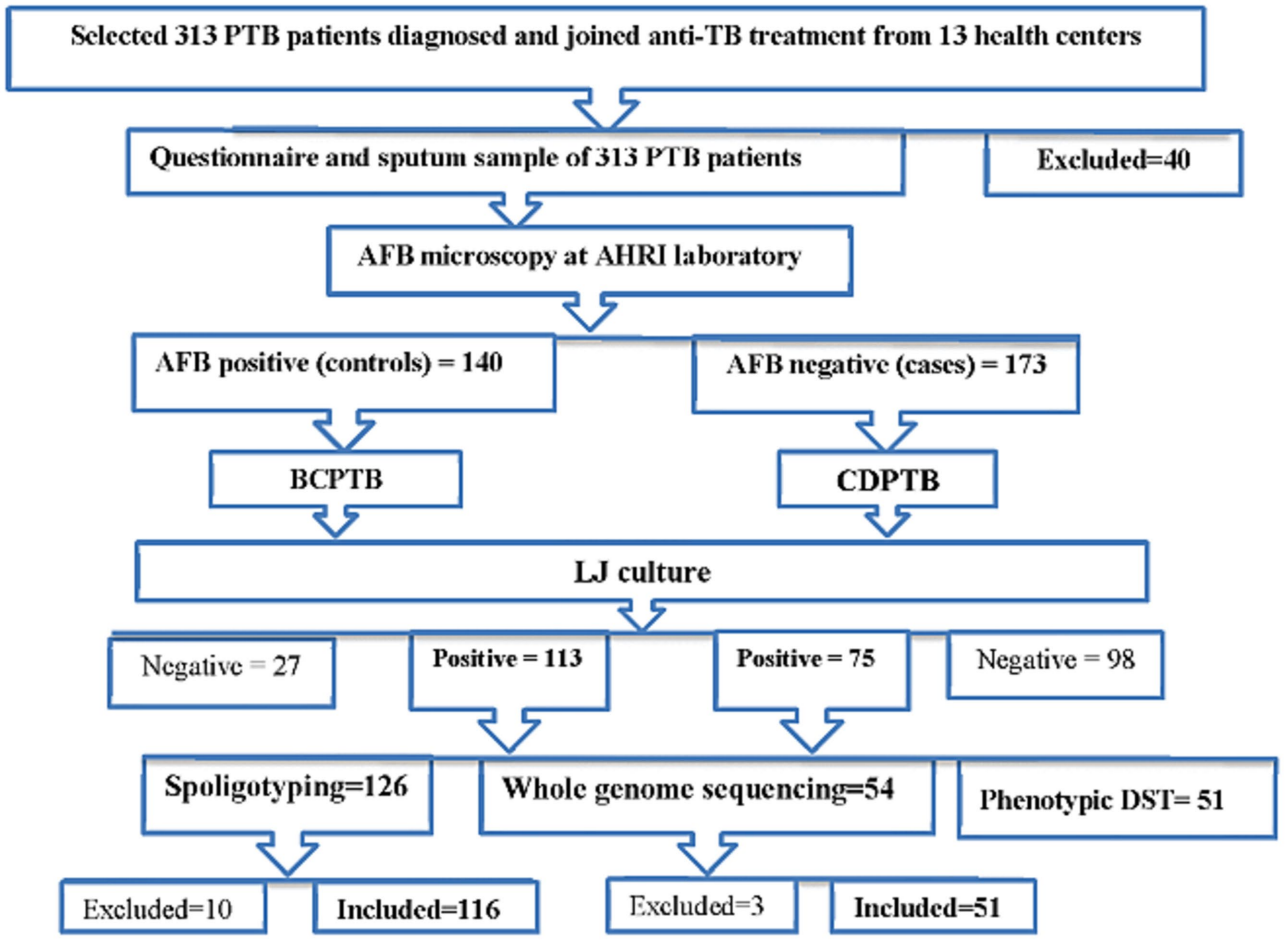
A flowchart mapped for patient classification and laboratory analysis procedure of the study, Addis Ababa, Ethiopia, 2021.

CDPTB patients (cases) were those who were clinically diagnosed as PTB at health facilities, based on the Ethiopian TB diagnostic guidelines (9) and confirmed AFB negative at the Armauer Hansen Research Institute (AHRI) TB laboratory using concentrated smear microscopy; whereas BCPTB (control group) were PTB patients diagnosed as bacteriologically confirmed PTB patients at the health facility and further confirmed AFB positive at AHRI laboratory by concentrated smear microscopy. A summary of patient recruitment and laboratory analysis procedure is shown in Figure 1.

### Sputum specimen collection and processing

Five to ten milliliters of sputum were collected from each patient and transported to the AHRI laboratory using a cold box. Upon arrival, samples were decontaminated using N-acetyl L-cysteine-sodium hydroxide (NALC-NaOH) and subjected to AFS and Lowenstein-Jensen (LJ) culture, if not done store appropriately at -20^0^c. All mycobacteriology laboratory safety procedures were followed as described in the global laboratory initiative biosafety manual (10) and internal standard operating procedures.

### *Mycobacterium tuberculosis* culture isolation and identification

Culture analyses were performed using LJ culture media following previously reported protocols (11, 12). Briefly, the NALC-NaOH treated sputum samples were centrifuged, and the sediment was inoculated onto an egg-based LJ culture medium. Two LJ medium tubes supplemented with glycerol were used for every sputum sample inoculation. All inoculated tubes were incubated at 37°C and growth of MTBC was checked weekly for 2 months. Typical MTBC colony morphology and subsequent AFS and Capilia neo-TB test (TANUS Laboratories, Japan) were used to confirm bacterial growth (13).

### Spoligotyping of MTBC isolates and database analyses

Heat-killed cells of LJ purely grown MTBC isolates (14) were spoligotyped according to previously described procedure (15). Briefly, PCR amplified products of each heat killed MTBC isolate using HotStarTaqMaster mix (Qiagen) and primers targeted to the Direct Repeat (DR) regions of MTBC (Thermofisher scientific, UK) were hybridized with a set of 43 immobilized oligonucleotides on a membrane. Then subsequently treated using the enhanced chemiluminescence method followed by exposure of the membrane to the X-ray film (Hyperfilm ECL, Amersham UK) (15). Finally, the hybridized bands developed on the film were visualized, verified, and entered into an Excel spread sheet for further analysis.

The verified spoligotype patterns, recorded on Excel spreadsheet, were converted into octal formats and compared with previously reported strains to define lineage, clades (family) and spoligotyping international type (SIT) (16) using the revised online MTBC molecular marker database SITVIT2 (SITVIT2 MTBC Genotyping Database (Pasteur-guadeloupe.fr)). The MTBC strains were considered as ‘orphans’ if a single strain was found in the SITVIT2 database and ‘new’ if their spoligotype pattern did not match with preexisting patterns in the database. The TB-Insight online tool, Run TB-Lineage^1^ particularly, the conformal Bayesian network (CBN), to assign major lineages (17) and Knowledge-Based Bayesian Networks (KBBN) (18), to assign clade or sub-family were used for those strains that were unidentified on the main website. MTBC strain clustering was considered if two or more strains harboring identical spoligotype pattern/SIT from different patients were identified in the study.

### Whole genome sequencing and bioinformatics analysis

#### DNA extraction and sequencing

The cetyltrimethyl-ammonium bromide (CTAB) method (19) was used to extract MTBC genomic DNA from heat killed fresh MTBC isolates grown on LJ solid culture media. The quality of extracted DNA was checked by gel electrophoresis and Nano drop 2000 (Thermo Fisher, Singapore). Then it was quantified by Qubit 4.0 fluorometer (Thermo Fisher, Singapore) and subjected to library preparation using Illumina library DNA Prep kit following the manufacturer’s protocol.^2^ Finally, libraries with qualified fragment sizes checked using Bioanalyzer 2,100 (Agilent, Germany) were pooled and subjected to WGS using illumina Nextseq500 sequencer (Illumina, Singapore) with paired-end read of 150 bp capacity.

#### Bioinformatics analysis

The quality of raw reads were checked using Fastqc tool, Fast QC (20) followed by trimming poor quality bases an adapters with a Trimmomatic (21) quality score of <20. All quality checks were combined and visualized with Multi QC v1.11 (22). Contamination check was done for all reads with Kraken 2 (23). Further analysis was performed for sequence reads ≥30 bp depth coverage and samples with >95% reference genome coverage using SAMtools (24). Duplicated reads were identified using Picard Mark Duplicates (25). Burrows-Wheeler Alignment Tool (BWA) (26) was used to align the raw paired end reads to the *M. tuberculosis* H37Rv reference genome (Gene bank accession number: NC000962.3). Variants were called using Free-Bayes (27) and annotated with snpEff (28) for further downstream analysis. Lineage and drug resistance were predicted using TB-Profiler (29). Using ancient *M. tuberculosis* (*M.canettii*) as root, Randomized Axelerated Maximum Likelihood (RaxML) with the general time reversal (GTR) model and 1,000 bootstrap value (30) were applied for SNP based phylogenetic reconstruction. Then Fig-tree^3^ was used to edit the generated tree. Moreover, WGS based clustering analysis was determined based on ≤12 SNPs difference whereas recent transmission index was based on ≤5 SNPs difference between the sequenced strains. MTBC strain discriminatory power of the genotyping methods were calculated using the online Hunter-Gaston Discrimination Index (HGDI) (31).

#### Phenotypic drug sensitivity testing

The *BD BACTEC* MGIT *960* system and MGIT SIRE DST kit, comprised of the four first-line anti-TB drugs including, rifampicin (RIF), isoniazid (INH), ethambutol (ETH) and, streptomycin (STN) a second line drug (32), was used for MTBC drug sensitivity testing according to the manufacturer instructions (33). Briefly, LJ grown MTBC isolates were inoculated into MGIT tubes, containing the drugs at a final WHO recommended critical concentration of 1.0 μg/mL for STR and RIF, 0.1 μg/mL for INH, and 5 μg/mL for ETH. Resistance to pyrazinamide (PZA) at 0.1 μg/mL final concentration was performed using the MGIT PZA drug kit separately according to the manufacturer’s instruction.

#### Quality assurance measures

Onsite training was given for data collectors before commencing the study. All laboratory analyses were performed according to the internal standard procedures and manufacturer instructions. Molecular grade water, *M. bovis* and *M. tuberculosis* H37Rv strains were used as a control for spoligotyping procedures. Moreover, a verification microscopy results for smear negative patients were done by a senior laboratory staff.

#### Data processing and statistical analysis

All laboratory data were organized, cleaned, and entered into SPSS version 25 (IBM, USA) and exported to Stata version 17 for analysis. Frequency and proportions were used to describe variables of interest. Chi-square was calculated to determine possible associations between study variables and a *p*-value <0.05 was considered statistically significant.

## Results

Out of the 313 PTB patients who participated in this study, 173 were CDPTB and 140 were BCPTB. The 56% patients participated in this study were male and age range from 18 years to 80 years with mean age 33.1 ± 12.4 years. Majority (86%) of patients came from urban areas and 27% of them were daily laborer (Supplementary Table 1).

Of the 313 sputum specimens collected from both patient groups, 180 (57.5%) grew on LJ culture media, of which a total of 116 MTBC isolates were spoligotyped, 42 from CDPTB and 74 from BCPTB patients. Distinct spoligotyping patterns were observed between clinically diagnosed and bacteriologically confirmed PTB patients.

**Figure 2:**
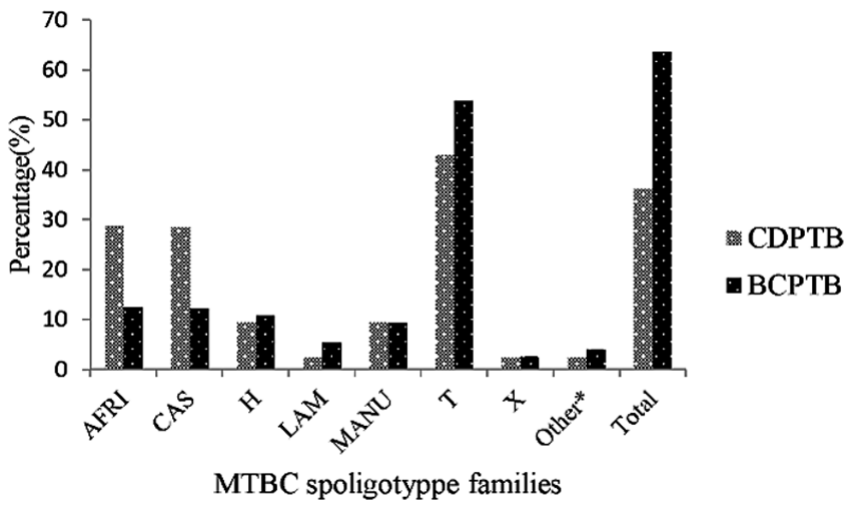
MTBC spoligotype family distribution among CDPTB and BCPTB patients, Addis Ababa, Ethiopia, 2021. *Spoligotype families/clades that include S, Zero, EAI7-BDG2, Cameroon and Unknown.

Four major MTBC lineages were identified using spoligotyping: the Euro-American lineage (lineage 4), East-African-Indian lineage (lineage 3), Indo-Oceanic lineage (lineage 1) and *M. africanum* (lineage 7 with WGS). In this paper the serial lineage naming of MTBC strains is applied to align with current similar publications (34). A relatively larger proportion of lineage 3 was observed among CDPTB patients while the proportion of lineage 4 was significantly larger among BCPTB patients (*p*-value <0.05; Table 1). Overall, 11 MTBC spoligotype families were identified from all PTB patients and no significant difference was noted between spoligotype families observed among CDPTB and BCPTB patients. The relative proportions of T family among CDPTB and BCPTB patients were 28.6 and 12.2%, respectively (*p*-value >0.05; Figure 2).

**Table 1:** Spoligotype based MTBC lineages among CDPTB and BCPTB patients, Addis Ababa, Ethiopia, 2021.

MTBC lineages	BCPTB	CDPTB	Total	*p*-value
	Number (%)	Number (%)	Number (%)	
Lineage 3 (East-African-Indian)	11 (14.9)	13 (31)	24 (20.69)	0.048			
Lineage 4 (Euro-American)	61 (82.4)	26 (62)	87 (75)
Other*	2 (2.7)	3 (7.14)	5 (4.31)
Total	74 (63.8)	42 (36.2)	116 (100)

*Other strains included; three lineage 1 (Indo-Oceanic) and two lineage 7 isolate. No: number.

**Figure 3:**
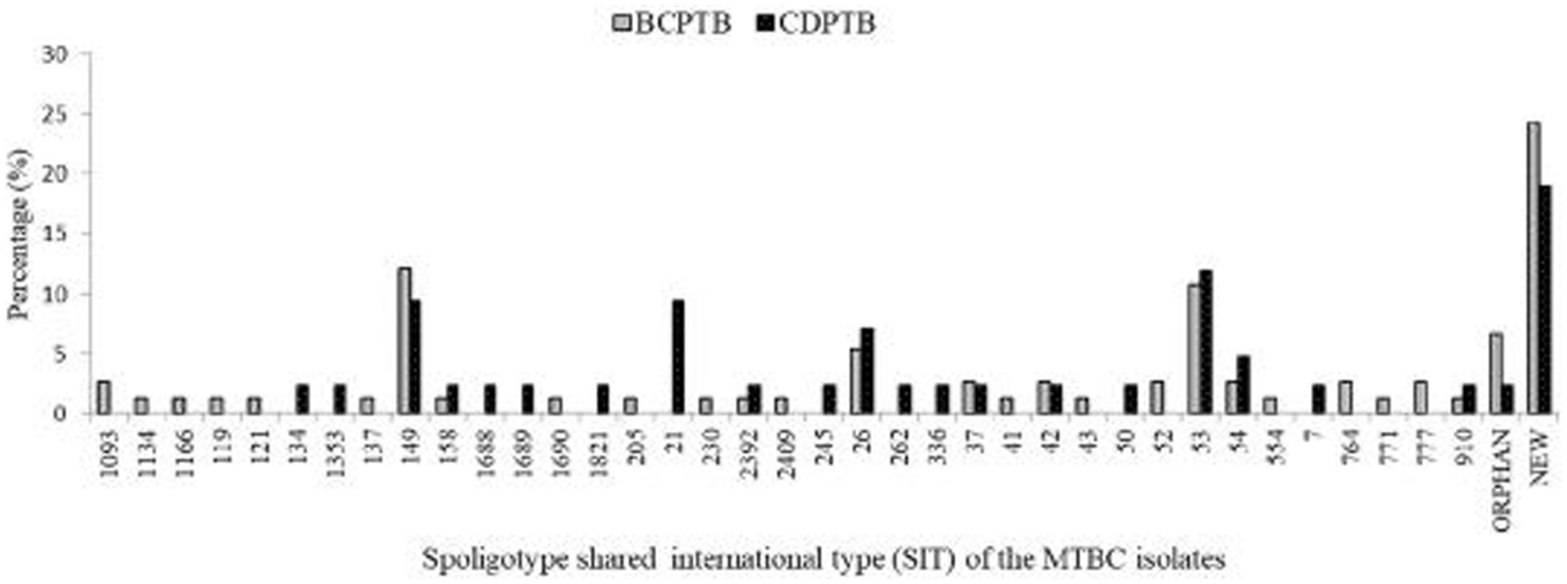
SIT of MTBC strains among CDPTB and BCPTB patients, Addis Ababa, Ethiopia, 2021.

Based on analysis of SIT among the 116 spoligotyped MTBC strains, 84 (72.4%) were pre-identified in the database. A total of 26 (22.4%) strains were identified as new, of which eight (30.8%) were from CDPTB patients and 18 (69.2%) from BCPTB patients. Moreover, six were orphans, of which four were from BCPTB patients (Figure 3). The highest cluster density size was observed among BCPTB patients within lineage 4 SIT 149 and SIT 53, each with 9 of 67 patients (Figure 3).

### WGS analysis of MTBC strains among CDPTB and BCPTB patients

Only 56 out of 188 culture confirmed MTBC isolates were randomly selected for WGS analysis Quality was checked for all the sequenced DNA including read length, GC content, and sequence quality; DNA sequence reads of two isolates were duplicates and three isolates were below 65% GC content and did not passed the quality check. The 51 samples that fulfilled the quality check were considered for downstream analysis, i.e., all the samples had an acceptable sequence quality score which was greater than 30 and read length between 119 bp and 138 bp (Supplementary Figure 1).

**Figure 4:**
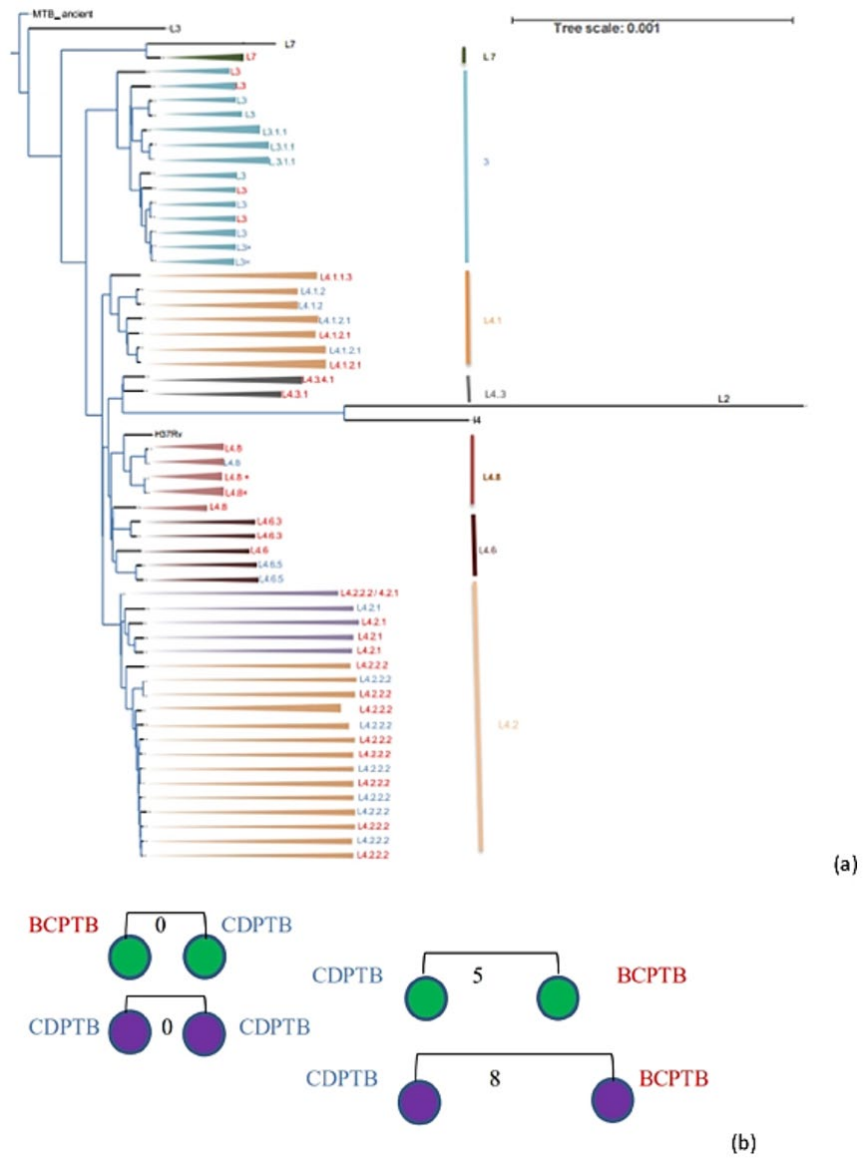
Phylogenetic tree (a) and clustered strains (b) of MTBC strains recovered from CDPTB and BCPTB patients, Addis Ababa, Ethiopia, 2021. Note: MTBC lineages colored blue are from CDPTB and red colored are from BCPTB patients, strains marked x are a duplicate; black branches are for M. canettii used to root for the phylogenetic tree construction and lineages used a control. Purple circles are lineage 3 and green circles are lineage 4; the numbers on the line indicates SNP distance.

Among the 51 MTBC isolates included in the genetic diversity and drug resistance analysis 72.54, 25.5 and 1.96% represented lineage 4, lineage 3 and lineage 7, respectively. While one isolate was identified as lineage 7 using WGS, it was labeled as *M. africanum* using spoligotyping. The MTBC strains in the three major lineages were further identified as 15 sublineages according to the constructed phylogenetic tree (Figure 4a) and compared between CDPTB and BCPTB patients. A different MTBC lineage and sublineage pattern and frequency was observed between CDPTB and BCPTB patients mirroring the results with spoligotyping. Lineage 3 was predominant among CDPTB 44.4% (8/18) compared to 15.5% (5/33) among BCPTB (*p*-value = 0.056). The proportion of Lineage 4 among BCPTB was also apparently larger 84.9% (28/33) compared to CDPTB, which was50% (9/18) (*p*-value = 0.107), shown in Table 2. Overall, the strain distribution among BCPTB and CDPTB was significantly different (*p*-value = 0.021).

**Table 2:** MTBC lineages among CDPTB and BCPTB patients based on WGS analysis, Addis Ababa, Ethiopia, 2021.

MTBC	PTB patient classification			
Lineages	BCPTB	CDPTB	Total	*p* –value
	Number (%)	Number (%)	Number (%)	
Lineage 3	5 (15.15)	8 (44.4)	13 (25.5)	0.021			
Lineage 4	28 (84.9)	9 (50)	37 (72.55)
Lineage 7	0	1 (5.5)	1 (1.96)
Total	33 (67.1)	18 (35.29)	51 (100)

We further compared the whole genome sequence patterns of MTBC after categorizing the isolates based on geneXpert diagnoses of the patients; the proportion of lineage 4 was relatively larger among geneXpert negative patients (80%) compared to geneXpert positive patients (60%) (*p*-value >0.05) (Table 3).

Among the sequenced MTBC isolates lineage 4 showed a more diversified strains than lineage 3 which is predominant among CDPTB patients (Figure 4a). The clustering analysis showed that 16% (8/51) of PTB patients had a clustered MTBC isolates based on ≤12 SNPs difference while about 8% (4/51) had a recent transmission based on ≤5 SNPs difference which was similar among CDPTB and BCPTB patients (Figure 4b). There was good agreement between WGS and spoligotyping in MTBC major lineage assignment in 98% (44/45) of the isolates; the discordance was observed in assignment of lineage 7 (Supplementary Table 2). The discriminatory power (DP) of the genotyping methods was calculated and both had a higher DP above 70%.

**Table 3:** WGS lineage distribution with respect to geneXpert test result among CDPTB and BCPTB patients, Addis Ababa, Ethiopia, 2021.

	BCPTB				CDPTB			
MTBC lineage type	geneXpert Positive	geneXpert Negative	Total	*p*-value	geneXpert positive	geneXpert Negative	Total	*p*-value
	No (%)	No (%)	No (%)		No (%)	No (%)	No (%)	
Lineage 3	5 (27.7)	0	5 (20.8)	0.449	6 (75)	2 (25)	8 (50)	0.037
Lineage 4	17 (77.3)	2 (100)	19 (79.2)		1 (12.5)	6 (75)	7 (43.8)	
Lineage 7	0	0	0		1 (12.5)	0	1 (6.3)	
Total	22 (81.7)	2 (8.3)	24 (100)		8 (50)	8 (50)	16 (100)	

### MTBC drug resistance pattern among CDPTB and BCPTB patients

In the current study, phenotypic drug susceptibility tests (DST) for first line anti-TB drugs; pyrazinamide (PZA), isoniazid (INH), rifampicin (RIF) and ethambutol (ETH), as well as the second-line drug streptomycin (STN), were performed for 51 MTBC isolates using the WGS analysis. More than 13.7% (7/51) of MTBC isolates were phenotypically resistant to one or more drugs. The proportion of MTBC isolates with any type of drug resistance was higher among CDPTB patients 16.7% (3/18) than among BCPTB patients 12% (4/33) (*p*-value >0.05) and a similar result was observed for MDR-TB, resistance to at least for RIF and INH (Table 4); however, these were not statistically significant. Phenotypic DST for PZA was done separately for 51 isolates and three PZA resistant MTBC strains including for one MDR-TB were detected in 3/51 (5.9%) and all of them were from CDPTB patients.

**Table 4:** Phenotypic and molecular drug resistance of MTBC isolates among CDPTB and BCPTB patients, Addis Ababa, Ethiopia, 2021.

DST result	Phenotypic method (MGIT)				Molecular method (WGS)			
	BCPTB	CDPTB	Total	*p* value	BCPTB	CDPTB	Total	*p*-value
	Number (%)	Number (%)	Number (%)		Number (%)	Number (%)	Number (%)	
MoDR	4 (12)	2 (11)	6 (12)	0.392	2 (6)	0	2 (4)	0.232
MDR	0	1 (5.6)	1 (1.96)		0	1 (5.6)	21 (2)	
DS	29 (88)	15 (83)	44 (86)		31 (35)	47 (65)	48 (92)	
Total	33 (65)	18 (35)	51 (100)		33 (65)	18 (35)	51 (100)	

MoDR, Mono-drug resistant; MDR, Multi-drug resistant; DS, Drug sensitive.

**Figure 5:**
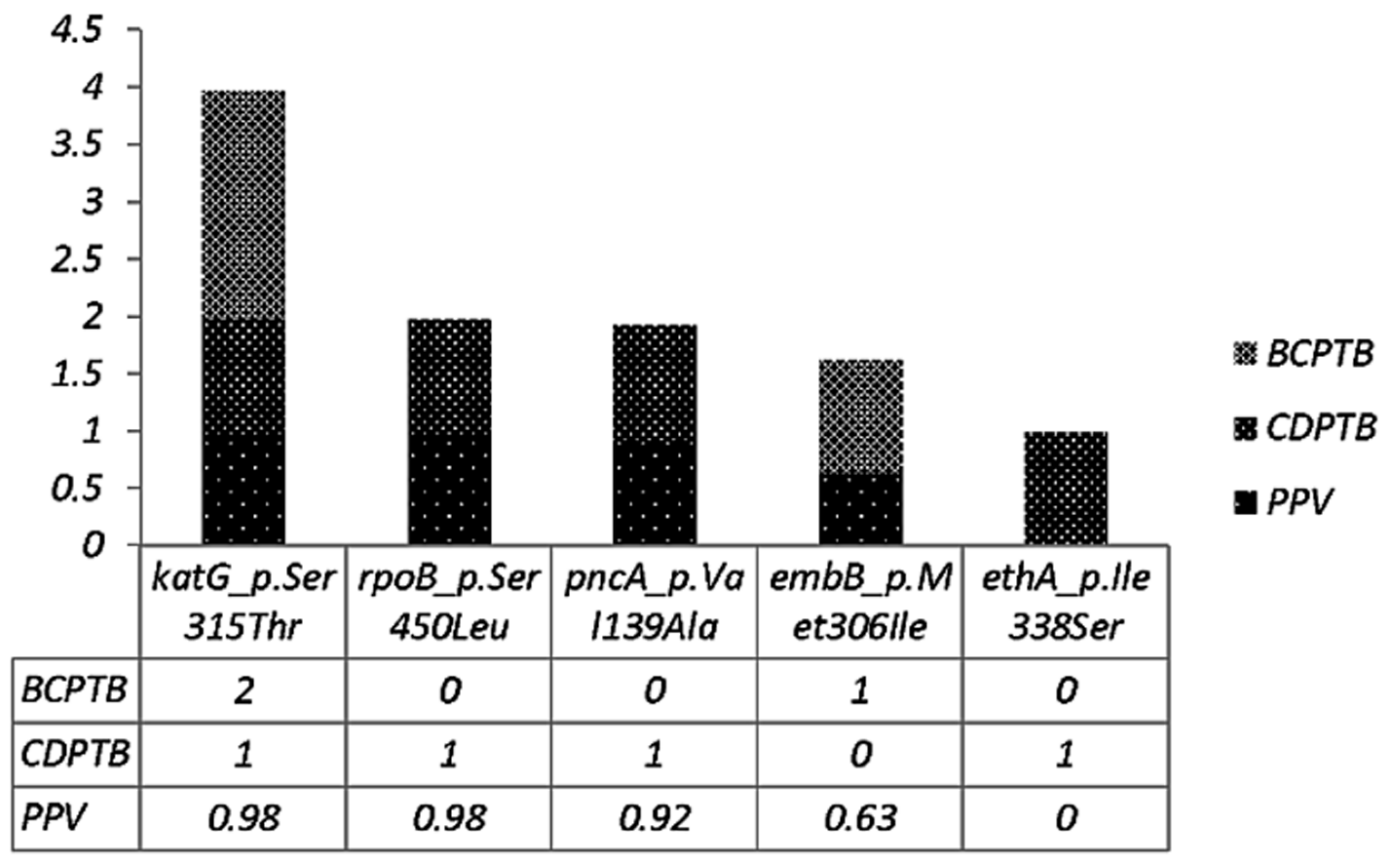
Drug resistance conferring mutations among CDPTB and BCPTB patients and their predictive value according to the WHO 2023 drug resistance mutation catalog.

The level of molecular drug resistance (as determined by WGS) among PTB patients in the current study was 5.9% (3/51) and considering the composite of all drugs, no significant difference was observed between CDPTB and BCPTB patients (*p*-value>0.05; Table 4). A single MTBC strain from CDPTB patient 5.6% (1/18) exhibited all the corresponding gene mutations conferring drug resistance for all the first-line anti-TB drugs and STN that aligns with the phenotypically detected DR. The other two molecular mono-resistant strains for INH 6% (2/33) were from BCTB patients. The known drug resistance mutations detected for RIF were *rpoB*_p.Ser450Leu and for INH, *katG*_p.Ser315Th. Strains with *katG* mutations had further ethanoamide drug resistance mutation *ethA_p.*Met1Arg 50% (2/3) including the MDR-TB patient (Table 5). A new *ethA_p.*Ile338Ser mutation was detected from a CDPTB patient that was resistant to all the first line anti-TB drugs (Table 5). All the mutations had more than 50% positive predictive value and were concordant with the updated WHO drug resistance mutation catalog (35) (Figure 5).

**Table 5:** WGS based drug resistance conferring mutations detected among PTB patients, Addis Ababa, Ethiopia, 2021.

Ser. no	Mutations detected for a particular anti-TB drugs					
	INH	RIF	PZA	ETH	STR	Ethanoamide
1	*katG_p.Ser315Thr*					*ethA_p.Met130Arg*
2	*katG_p.Ser3154Thr*	*rpoB_p.Ser450Leu*	*pncA_p.Val139Ala*	*embB_p.Met306Ile*	*rpsL_p.Lys43Arg*	*ethA_p.Ile338Ser*
3	*katG_p._Ser315Thr*					

A discordant result was also found in drug resistance detection by phenotypic and molecular methods. Any known mutations, including on *panA* gene were not detected by WGS for the two phenotypically PZA mono-resistant strains from CDPTB patients. Similarly mutations on *katG* gene for phenotypically INH mono-resistant strains from another two BCPTB patients were not detected. A mono-drug resistant *katG* mutation from a BCPTB patient was not detected by the phenotypic method. All the mutations are listed in the revised WHO catalog, except the mutation for ethanoamide drug resistance (*ethA*_p.Ile338Ser) (35).

## Discussion

In the present study, we evaluated the distribution of MTBC strains among PTB patients with either bacteriologically confirmed pulmonary TB (smear positive TB) or clinically diagnosed pulmonary TB (smear negative TB). We observed that the overall lineage distribution among all pulmonary TB patients was similar with other genotypic studies in Ethiopian pulmonary TB patients with a predominance of lineage 4 and lineage 3 strains (36–39). The MTBC lineage distribution among BCPTB patients and among CDPTB patients coincided with the overall distribution among PTB patients however, in a comparative analysis of the MTBC lineage distribution among CDPTB and BCPTB, a significantly larger proportion of lineage 3 MTBC strain was observed among CDPTB patients than among BCPTB patients, in which lineage 4 was a predominant MTBC strain. The laboratory detection of MTBC bacilli particularly AFS smear microscopy is usually associated with TB diseases severity which is manly characterized by lung cavitary lesions (5). Cavitary TB, on the other hand, was shown to be associated with MTBC lineages 4 (40) while in another study it was associated with lineage 3 (41). In a recent study, no association was observed between MTBC lineage 4 and 3 with cavitary TB (42).

The study findings imply that lung cavitary lesion is the result of different factors including the host immune response, MTBC genotype or underlying other diseases (43). It might be useful to consider the difference in mycobacterial genes related to lipid metabolism among MTBC strains for the MTBC lineage difference between CDPTB and BCPTB patients; for example lineage 3 tend to be AFB negative on staining more frequently than lineage 4 strains, since it is characterized by inherently more abundant proteins involved in lipid metabolism than lineage 4 and lineage 7 (7), which possibly interfere with penetration of the acid-fast staining reagent that may precludes ease of laboratory detection than lineage 4. The observed differences in MTBC strain predilections between CDPTB and BCPTB patients could also be alluded to challenges related to bacterial diagnoses among CDPTB patients. However, further studies are required on this to better understand MTBC lineages in relation with different TB different TB laboratory diagnostic outcomes.

The comparable clustering rate with the recent TB transmission index among CDPTB and BCPTB, defined based on the WGS data, in the current study might infer delayed TB diagnosis and treatment in CDPTB patients (44). The current study finding is similar with other study on a similar group of patients (45), while higher than recent study report on the diseases transmission potential of CDPTB patients (46). Though the sample size, patient classification method and the study design contributes for study report differences, the current finding may indicate the importance of applying BCPTB patient management practices to control and prevent infection similar for CDPTB patients. CDPTB patients are often considered less contributors to TB disease transmission and often attracts less attention by health personnel’s as well as TB control programs (47). This study emphasizes the need for understanding of contributions of MTBC strain diversity and drug resistance profiles in CDPTB patients and strengthen the proper case management of patients as well as the TB control program to curb TB transmission in the community. On the other hand, defining transmissions rates related to molecular strains may ultimately depend on prospective studies with molecular evaluation of both index cases and contacts with larger sample size.

The proportion of resistance to any of anti-TB drug as well as multi-drug resistance was lower than a recent study conducted in Addis Ababa among CDPTB patients (3). The observed difference across the studies could be attributed to DR detection method (MGIT vs. LJ), study period, or method used for PTB patient classification (direct vs. concentrated smear microscopy). Remarkably, PZA resistance was significantly higher among CDPTB than BCPTB patients. To our knowledge, this is the first report of such resistance in Ethiopia among CDPTB patients; however, an increased proportion of PZA resistance among CDPTB has been reported in a study from China (48). Differences in drug resistance between CDPTB and BCPTB warrants further investigation with larger samples sizes.

The most frequent gene mutations associated with INH and RIF resistance were similar with other study report (49). The newly detected *ethA*_p.Ile338Ser mutation on lineage 3 isolate from both CDPTB and geneXpert rifampicin resistance (RR) TB negative patient, whom resistant for all the first-line anti-TB drugs implies the possibility of extensively DR-TB in this group of patients that warrants future studies including both first and second-line drug resistance profile among MTBC strains with larger sample size.

Recently, drug resistance mutations has been the target for WGS and a research focus to bring into the routine diagnosis since culture based DST and the molecular methods like geneXpert have limitations in detecting emerging resistant strains. In our study, all the mutations were in agreement with the detected phenotypic drug resistance and have more than 50% positive predictive, values, urging the universal DST (35), including for CDPTB patients. However, the undetected molecular resistance on known drug resistant gene for the phenotypic DR strains could be due to the other MTBC drug resistance mechanisms including the intrinsic ones like efflux pump, mutations in gens coding for important cell wall proteins PE II and others (50). A similar findings were also reported previously (51). This study attempted to compare the MTBC genotype between study groups with PTB laboratory diagnosis outcomes, including geneXpert test results, which is the first in the study area. However, there are limitations that may impact the study output, inherent to the nature of the study design (unmatched case–control) and the lower number of isolates used for drug susceptibility testing which could possibly impact the expected associations. However, these can be partly compensated using the most robust genotyping method WGS. In addition we used concentrated AFS smear microscopy for patient diagnoses, positivity impacting the quality of laboratory testing and increasing in the identification of more CDPTB patient into the study.

In summary, our results suggest that there are differences in underlying MTBC strain diversity among CDPTB and BCPTB patients. These strain differences may contribute to differences in underlying clinical presentations, challenges in the laboratory identification of CDPTB patients, and differences in drug resistance. These observations warrant further investigation of the sequenced isolates as well as additional studies with variables which are not addressed in this study including more sensitive respiratory specimens such as bronchoalveolar lavage and induced sputum for better recommendation.

## Data Availability

The original contributions presented in the study are included in the article/Supplementary material, further inquiries can be directed to the corresponding author. Sequence data is available at NCBI (SRA) BioProject ID: PRJNA1182313.

## Footnotes

^1^ http://tbinsight.cs.rpi.edu/run_tb_lineage.html

^2^ https://support.illumina.com/content/dam/illuminasupport/documents/documentation/chemistry_documentation/illumina_prep/illumina-dna-prep-reference-guide-1000000025416-09.pdf

^3^ https://github.com/rambaut/figtree/releas
