# Supplementary material for: Genotypes and drug resistance pattern of Mycobacterium tuberculosis complex among clinically diagnosed pulmonary tuberculosis patients
Source: Front Public Health. 2024 Dec 2;12:1420685. doi: 10.3389/fpubh.2024.1420685 (PMC11646991; doi:10.3389/fpubh.2024.1420685)
Supplement: Supplementary file 1 [file Data_Sheet_1.PDF]

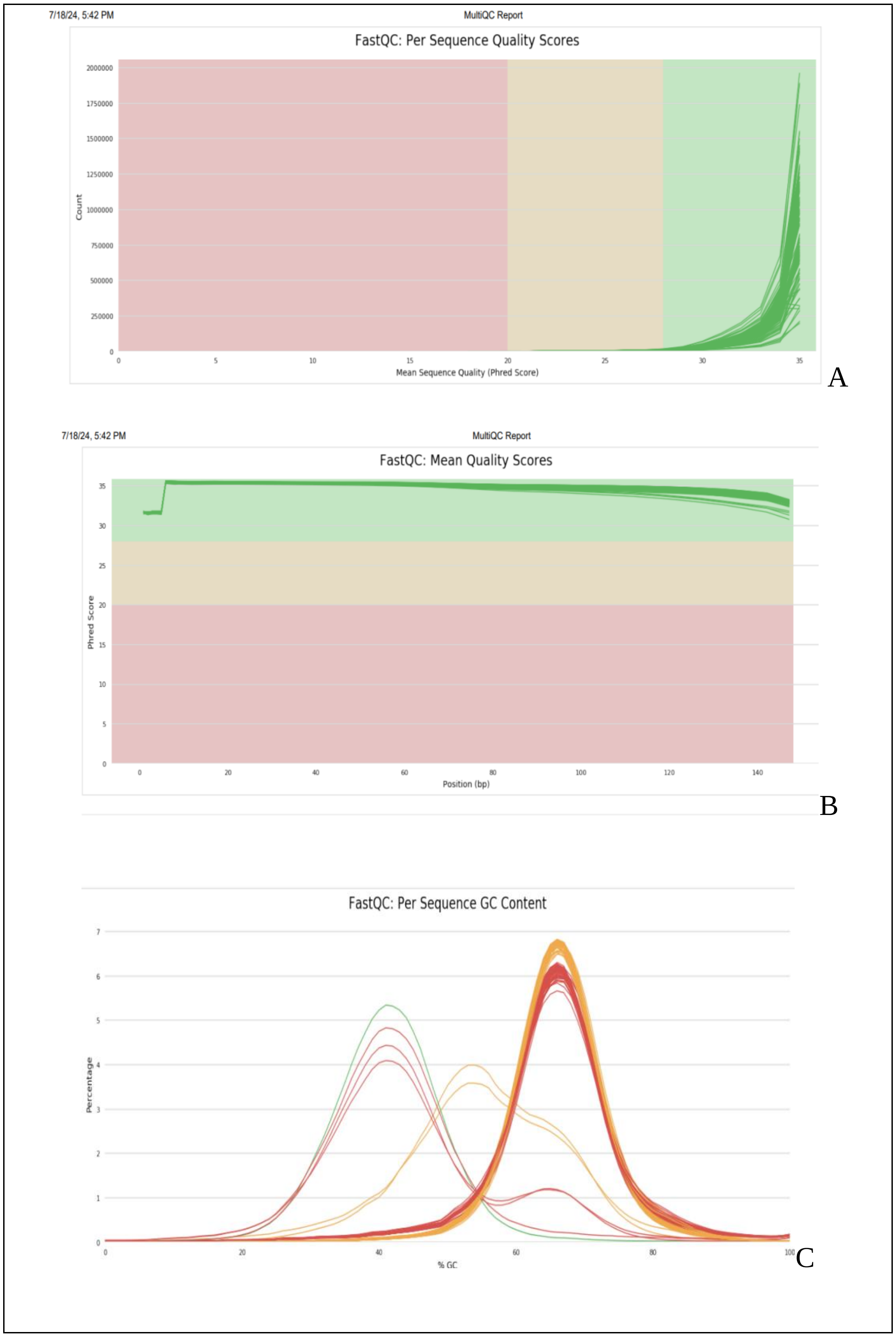

Supplementary Figure 1. Sequence quality score plots of the 56 MTBC isolates, Addis Ababa, 2021. (A: the quality score per sequence B: mean sequence quality score, C: G-C content.)
