# Supplementary material for: Genotypes and drug resistance pattern of Mycobacterium tuberculosis complex among clinically diagnosed pulmonary tuberculosis patients
Source: Front Public Health. 2024 Dec 2;12:1420685. doi: 10.3389/fpubh.2024.1420685 (PMC11646991; doi:10.3389/fpubh.2024.1420685)
Supplement: Supplementary file 2 [file Table_1.DOCX]

Supplementary Table 2 Spoligotyping and whole genome sequencing data of CDPTB and BCPTB patients, Addis Ababa, Ethiopia, 2021

| **Ser. No** | **WGS lineages** | **Spoligotyping lineages** | | | **PTB type** |
| --- | --- | --- | --- | --- | --- |
|  |  | SIT | family/ clade | Lineage name |  |
|  | Lineage 4.2.2.2 | 53 | T | EA | BCPTB |
|  | Lineage 3 | 26 | CAS1-DELHI | EAI | BCPTB |
|  | Lineage 4.2.1 | 53 | T | EA | CDPTB |
|  | Lineage 4.2.2.1 | 42 | LAM7-TUR(Turkey) | EA | BCPTB |
|  | Lineage 4.2.2.2 | 53 | T | EA | BCPTB |
|  | Lineage 4.1.2 | 53 | T | EA | CDPTB |
|  | Lineage 3.1.1 | ORPHAN | CAS1-DELHI | EAI | BCPTB |
|  | Lineage 4.2.2.2 | 134 | H3 | EA | CDPTB |
|  | Lineage 4.3.1 | ORPHAN | Cameroon | EA | BCPTB |
|  | Lineage 3 | 2392 | CAS1-DELHI | EAI | CDPTB |
|  | Lineage 4.8 | 53 | T | EA | CDPTB |
|  | Lineage 4.2.2.2 | 149 | T3_ETH | EA | CDPTB |
|  | Lineage 4.6.3 | 53 | T | EA | BCPTB |
|  | Lineage4.8 | 262 | H3-Ural-1 | EA | BCPTB |
|  | lineage4.6 | 554 | T2 | EA | BCPTB |
|  | lineage 4.2.2.2 | 149 | T3_ETH | EA | CDPTB |
|  | lineage3 | NEW | CAS1-DELHI | EAI | CDPTB |
|  | lineage 4.1.2.1 | 50 | H3 | EA | CDPTB |
|  | lineage 4.2.2.2 | 336 | X1 | EA | CDPTB |
|  | lineage 3.1.1 | 21 | CAS1-KILL | EAI | CDPTB |
|  | lineage 4 | 7 | T | EA | CDPTB |
|  | lineage 7 | 910 | AFR | MA | CDPTB |
|  | lineage3 | NEW | CAS1-DELHI | EAI | CDPTB |
|  | lineage4.2.2.2 | 119 | X1 | EA | BCPTB |
|  | lineage4.2.2.2 | 149 | T3_ETH | EA | BCPTB |
|  | lineage 4.2.2.2 | 149 | T3_ETH | EA | CDPTB |
|  | lineage4.1.2.1 | 54 | MANU2 | EA | BCPTB |
|  | lineage4.1.1.3 | NEW | MANU2 | EA | BCPTB |
|  | lineage4.6 | NEW | T1-RUS2 | EA | BCPTB |
|  | lineage 4.1.2.1 | 121 | H3 | EA | BCPTB |
|  | lineage4.2.1 | 1134 | H3-Ural-1 | EA | BCPTB |
|  | lineage3 | NEW | MANU2 | EA | BCPTB |
|  | lineage4.2.2.2 | 52 | T2 | EA | BCPTB |
|  | lineage4.3.4.1 | 771 | T | EA | BCPTB |
|  | lineage3 | NEW | CAS1-DELHI | EAI | BCPTB |
|  | lineage4.1.2 | ORPHAN | H37Rv(T) | EA | BCPTB |
|  | lineage 3.1.1 | 21 | CAS1-KILL | EAI | CDPTB |
|  | lineage4.6.3 | 2409 | AMBIGIOUS; T3, T5 | EA | BCPTB |
|  | lineage4.2.2.2 | 149 | T3_ETH | EA | BCPTB |
|  | lineage4.2.2.2 | 149 | T3_ETH | EA | BCPTB |
|  | lineage4.8 | NEW | T | EA | BCPTB |
|  | lineage4.2.1 | 53 | T | EA | BCPTB |
|  | lineage 3 | NEW | MANU2 | EA | BCPTB |
|  | lineage4.2.2.2 | 52 | T2 | EA | BCPTB |

^EA: Euro American, EAI East African Indian, MA;^ *^M. africanum^*

^CDPTB: smear negative PTB, BCPTB: smear positive PTB^
