# Supplementary material for: Genotypes and drug resistance pattern of Mycobacterium tuberculosis complex among clinically diagnosed pulmonary tuberculosis patients
Source: Front Public Health. 2024 Dec 2;12:1420685. doi: 10.3389/fpubh.2024.1420685 (PMC11646991; doi:10.3389/fpubh.2024.1420685)
Supplement: Supplementary file 3 [file Table_2.DOCX]

Supplementary Table1. Socio-demographic characteristics and TB related factors among SNPTB and SPPTB patients, Addis Ababa, Ethiopia, 2021

| **Study variables** | | **SPPTB** | | **SNPTB** | | **Total** | |  |
| --- | --- | --- | --- | --- | --- | --- | --- | --- |
|  |  | **Freq.** | **%** | **Freq.** | **%** | **Freq.** | **%** |  |
| **Gender** | Female | 49 | 44.6 | 89 | 44 | 93 | 56 |  |
|  | Male | 61 | 55.6 | 114 | 56 | 175 | 44 |  |
| **Age group (years)** | ≥ 45 | 19 | 14.0 | 41 | 24.6 | 60 | 19.8 |  |
|  | <45 | 117 | 86.0 | 126 | 75.4 | 243 | 80.2 |  |
| **Educational level** | Literate | 109 | 81.3 | 113 | 69.3 | 222 | 74.7 |  |
|  | Illiterate | 25 | 18.7 | 50 | 30.7 | 75 | 25.3 |  |
| **Occupation** | Daily laborer | 45 | 33.3 | 36 | 21.8 | 81 | 27.0 |  |
|  | Non-daily laborer | 90 | 66.7 | 129 | 78.2 | 219 | 73.0 |  |
| **Alcohol use** | Yes | 41 | 31.1 | 37 | 22.4 | 78 | 26.3 |  |
|  | No | 91 | 68.9 | 128 | 77.6 | 219 | 73.7 |  |
